# Circulating Concentrations of Cathelicidin Anti-Microbial Peptide (CAMP) Are Increased during Oral Glucose Tolerance Test

**DOI:** 10.3390/ijms241612901

**Published:** 2023-08-17

**Authors:** Alexandra Höpfinger, Thomas Karrasch, Andreas Schäffler, Andreas Schmid

**Affiliations:** Department of Internal Medicine III, Giessen University Hospital, Klinikstrasse 33, 35392 Giessen, Germany; thomas.karrasch@innere.med.uni-giessen.de (T.K.); andreas.schaeffler@innere.med.uni-giessen.de (A.S.); andreas.schmid@innere.med.uni-giessen.de (A.S.)

**Keywords:** Cathelicidin antimicrobial peptide, CAMP, adipokine, oral glucose tolerance test, OGTT, adipocyte

## Abstract

Recent investigation has revealed the significant role of Cathelicidin antimicrobial peptide (CAMP) in infection defense and innate immunity processes in adipose tissue. Meanwhile, knowledge of its regulation and functions in metabolic contexts as an adipokine remains sparce. The present study investigated the postprandial regulation of circulating CAMP levels during oral glucose tolerance tests (OGTTs). Eighty-six metabolically healthy volunteers participated in a standardized 75 g-2 h-OGTT setting. The effects of exogenous glucose, insulin, and incretins on *CAMP* expression in human adipocyte culture (cell-line SGBS) were studied in vitro. CAMP concentrations in blood serum samples were measured by ELISA techniques and adipocyte gene expression levels were quantified by real-time PCR. Of note, base-line CAMP serum quantities were negatively correlated with HDL cholesterol levels as well as with the anti-inflammatory adipokine adiponectin. During the 2 h following glucose ingestion, a significant rise in circulating CAMP concentrations was observed in considerable contrast to reduced quantities of fatty acid binding proteins (FABP) 2 and 4 and dipeptidyl peptidase 4 (DPP4). In SGBS adipocytes, neither differing glucose levels nor insulin or incretin treatment significantly induced *CAMP* mRNA levels. According to our data, glucose represents a positive postprandial regulator of systemic CAMP. This effect apparently is not mediated by the regulatory impact of glucose metabolism on adipocyte *CAMP* expression.

## 1. Introduction

Obesity represents one of the worldwide major public health issues with a sustained rise in prevalence and severity [1]. In concurrence with its comorbidities—collectively being referred to as metabolic syndrome [2]—it adversely affects quality of life [3] and life expectancy [4]. Of particular importance, morbid obesity is associated with adipose tissue malfunction (adipoflammation) and a chronic state of low-grade, sterile inflammation (metaflammation) [5,6,7], favoring the onset of peripheral insulin resistance and type 2 diabetes mellitus (T2D). Characterizing molecular mechanisms linking metabolic dysregulation to immune modulation, especially in adipose tissue, therefore represents a valuable objective of the current investigation.

The physiological relevance of the immunological processes in adipose tissue has increasingly been recognized during recent years. Numerous immunomodulatory functions are exerted by secretory proteins from adipose tissue, so-called adipokines [8]. Among these very heterogenous factors, adipose-derived Cathelicidin antimicrobial peptide (CAMP; also: LL37, CAP-18) has gained particular attention due to its crucial role in host defense against subdermal infection [9]. Previous investigation has further elucidated its regulation in adipose tissue and adipocyte innate immunity [10,11]. Of note, a recent study on a cohort of morbidly obese individuals undergoing weight loss therapy revealed circulating CAMP to be induced upon bariatric surgery, potentially involving effects mediated by systemic bile acids and incretins [12].

The postprandial dynamics of circulating CAMP as well as the regulation of adipocyte *CAMP* gene expression by factors of carbohydrate metabolism represent crucial issues for the development of molecular therapeutic options targeting metaflammation and associated morbidities. However, knowledge regarding these aspects remains sparce so far.

Fatty acid binding proteins (FABP), acting as chaperone molecules in cellular lipid distribution, play a crucial role in multiple processes of lipid metabolism [13]. Adipocyte-derived FABP4 has early been identified as a key factor in obesity-associated adipose inflammation and insulin resistance [14,15] and has previously been reported to decrease during oral glucose tolerance tests (OGTTs) [16]. The intestinal fatty acid binding protein (FABP2) is expressed in the enterocytes of the small intestine, where it has been assumed to primarily support apical fatty acid import from the intestinal lumen [17]. Of note, genetic studies revealed an association of A54T missense mutation with impaired glucose tolerance [18], indicating the significant role of this FABP in postprandial glucose homeostasis and insulin action.

The postprandial elevation of systemic glucose levels involves a concomitant rise in circulating incretins such as glucose-dependent insulinotropic polypeptide (GIP) and glucagon-like peptide-1 (GLP-1) [19]. Dipeptidyl peptidase-4 (DPP4) activity is crucial for GIP and GLP-1 degradation in order to maintain incretin homeostasis [19].

Therefore, the present study focused on

- An investigation of the short-term effects of oral glucose ingestion on systemic CAMP levels in vivo, alongside FABP2/4 and DPP4 as significant factors of postprandial metabolic regulation;
- The characterization of glucose, insulin, and incretins regarding their ability to modulate human adipocyte *CAMP* expression in vitro.

## 2. Results

### 2.1. Characteristics of the Study Cohort

Among the 86 healthy participants of the study (57 females, 29 males), 40 had a normal weight (BMI < 25 kg/m^2^) and 46 were overweight or obese (BMI ≥ 25 kg/m^2^). The base-line anthropometric and biochemical characteristics of the cohort are summarized in Table 1.

The mean CAMP serum concentration at the study base-line was 28.08 ± 14.45 ng/mL (range: 4.15–76.00 ng/mL). Slight and non-significant differences were observed between male and female subjects (31.49 ± 15.00 ng/mL vs. 26.34 ± 13.97 ng/mL) and between individuals with a normal weight (BMI < 25 kg/m^2^) and overweight individuals (BMI ≥ 25 kg/m^2^) (25.06 ± 13.15 ng/mL vs. 30.71 ± 15.13 ng/mL). A more detailed analysis regarding BMI stratification also did not reveal significant differences in systemic CAMP quantities for the 5th and 95th percentiles. Of note, a minor proportion of subjects (n = 7) exhibited considerably high triglyceride levels (>200 mg/dL) at study base-line, indicating a possible prediabetic state. The base-line CAMP levels of these individuals were significantly elevated when compared to the remaining study cohort (46.98 ± 14.34 ng/mL vs. 26.41 ± 13.29 ng/mL; *p* = 0.001).

Of note, there was a significant sexual dimorphism, with men exhibiting higher base-line DPP4 levels than women (524.19 ± 103.96 ng/mL vs. 468.68 ± 105.44 ng/mL; *p* = 0.025). On the contrary, elevated FABP4 concentrations were observed in females when compared to male individuals (5261.68 ± 4503.67 pg/mL vs. 3982.63 ± 2831.50 pg/mL; *p* = 0.016).

### 2.2. Correlations of Serum CAMP Levels with Anthropometric and Biochemical Parameters

A correlation analysis applying a non-parametric Spearman-rho test revealed significant correlations of base-line systemic CAMP concentrations with HDL cholesterol, adiponectin, and lipocalin-2 (Table 2). The results of correlation analysis of systemic CAMP concentrations and parameters of glucose and lipid metabolism at study base-line are shown in Supplementary Table S1.

**Figure 1:**
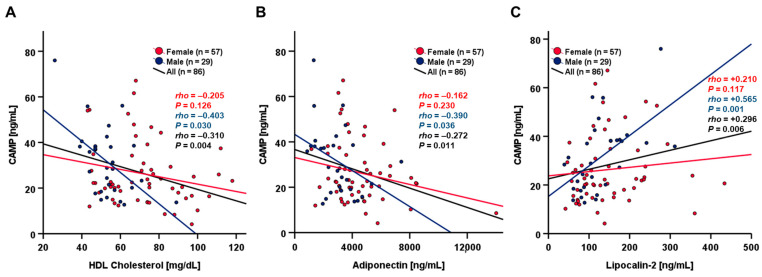
Correlations of base-line CAMP serum concentrations with metabolic and biochemical parameters. CAMP serum concentrations correlate significantly negatively with HDL cholesterol (**A**) and Adiponectin (**B**) in the entire study cohort and in males, but not in females. CAMP serum concentrations correlate significantly positively with Lipocalin-2 in the entire study cohort and in males, but not in females (**C**). Serum protein levels of adipokines were quantified by ELISA. HDL cholesterol quantities were assessed by routine laboratory methods. HDL, high-density lipoprotein cholesterol.

Of note, a substantial divergence between males and females was observed regarding the significant correlations of CAMP with HDL cholesterol, adiponectin, and lipocalin-2 (Figure 1). Negative correlations with HDL cholesterol and adiponectin levels were observed exclusively in males and were absent in females (Figure 1A,B). Similarly, only male subjects exhibited a significant positive correlation of CAMP and lipocalin-2 concentrations (Figure 1C). For the entire study cohort, the strong correlations of CAMP with HDL cholesterol (*rho* = −0.336, *p* = 0.002) and lipocalin-2 levels (*rho* = +0.257, *p* = 0.017) persisted throughout the OGTT after 2 h (Supplementary Table S2).

### 2.3. Dynamics of Systemic CAMP, FABP2/4 and DPP4 Concentrations during OGTT

**Figure 2:**
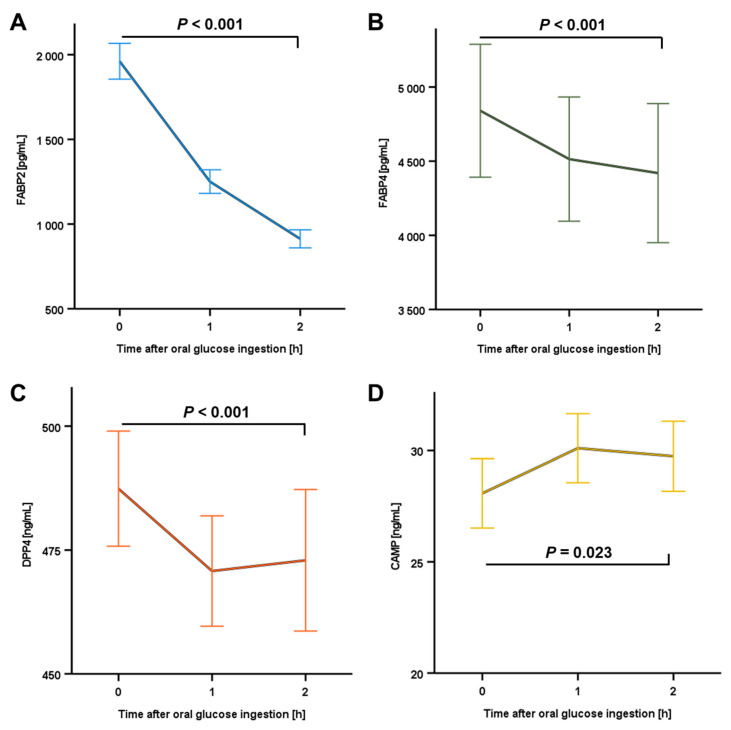
Circulating concentrations of FABP2 (**A**), FABP4 (**B**), DPP4 (**C**), and CAMP (**D**) during OGTT. Serum protein levels were quantified by ELISA and means are expressed ± standard error of the mean (SEM). CAMP, Cathelicidin antimicrobial peptide; DPP4, dipeptidyl peptidase-4; FAPB, fatty acid binding protein.

Oral glucose ingestion (75 g) resulted in an immediate and substantial decline of circulating FABP2 concentrations from 1961 to 913 pg/mL during the OGTT (*p* < 0.001) (Figure 2A). The serum levels of FABP4 (from 4841 to 4420 pg/mL; *p* < 0.001) and DPP4 (from 487.4 to 472.9ng/mL; *p* < 0.001) exhibited a rather moderate yet statistically significant decrease (Figure 2B,C). These observed dynamics in circulating FABP2, FABP4, and DPP4 protein quantities were independent of sexual dimorphism and differences in BMI.

In contrast, a significant increase in CAMP serum levels occurred throughout 2 h, with a rise in mean concentration from 28.08 ng/mL to 29.74 ng/mL within the whole study cohort (Figure 2D). Of note, a subgroup analysis revealed that the increase upon glucose ingestion was significant in female (n = 57; *p* = 0.045) but not in male individuals (n = 29; *p* = 0.485), indicating a potential sexual dimorphism. Furthermore, we observed a significant increase (*p*= 0.008) in CAMP serum levels throughout 2 h exclusively in normal-weight individuals (n = 40) but not in overweight study subjects (n = 46) (Supplementary Figure S1).

### 2.4. Effects of Glucose, Insulin and Incretins on Adipocyte Gene Expression In Vitro

**Figure 3:**
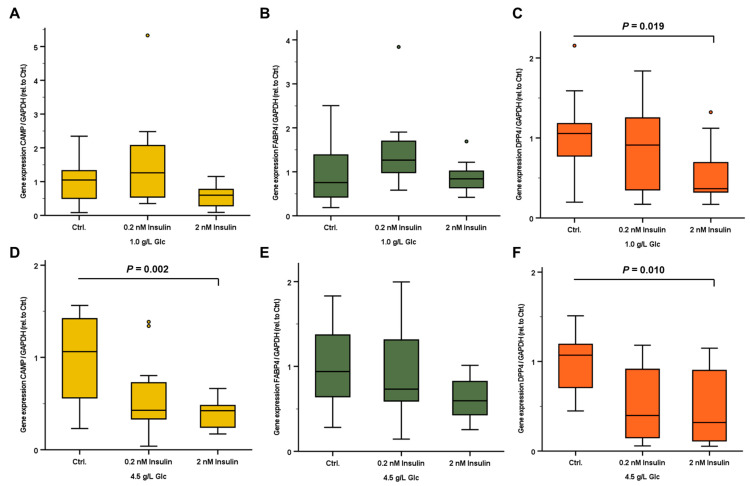
Relative gene expression levels of *CAMP*, *FABP4*, and *DPP4* in human mature SGBS adipocytes. Cells were treated with 0.2 and 2 nM insulin under either physiological (1.0 g/L Glc; (**A**–**C**)) or supra-physiological (4.5 g/L Glc; (**D**–**F**)) glucose concentrations. *CAMP*, *FABP4*, and *DPP4* mRNA concentrations were quantified by real-time PCR and were normalized to *GAPDH* expression. CAMP, Cathelicidin antimicrobial peptide; DPP4, dipeptidyl peptidase-4; FAPB4, fatty acid binding protein 4; GAPDH, glyceraldehyde-3-phosphate dehydrogenase.

Considering the observed physiological impact of glucose ingestion, in vitro experiments were applied in order to identify and characterize potential direct effects of glucose on adipocyte gene expression of *CAMP*, *FABP4*, and *DPP4*. In human mature SGBS adipocytes cultured in medium containing moderate glucose concentration (5.6 mM/1.0 g/L), overnight treatment (18 h) with insulin resulted in a significant decrease in *DPP4* mRNA concentrations whereas *CAMP* and *FABP4* expression remained unchanged (Figure 3A–C).

An insulin stimulation under the conditions of supraphysiological glucose concentration (25 mM/4.5 g/L) resulted in decreased *CAMP* and *DPP4* expression levels without significantly affecting the *FABP4* mRNA concentrations (Figure 3D–F).

**Figure 4:**
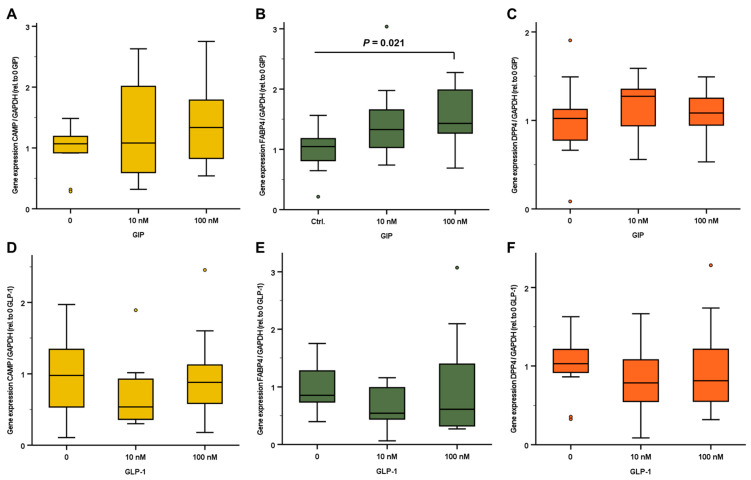
Relative gene expression levels of *CAMP*, *FABP4*, and *DPP4* in human mature SGBS adipocytes. Cells were treated with 10 nM and 100 nM of either GIP (**A**–**C**) or GLP-1 (**D**–**F**) in culture medium containing moderately elevated glucose concentration (3.151 g/L). *CAMP*, *FABP4*, and *DPP4* mRNA concentrations were quantified via real-time PCR and were normalized to *GAPDH* expression. CAMP, Cathelicidin antimicrobial peptide; DPP4, dipeptidyl peptidase-4; FAPB4, fatty acid binding protein 4; GAPDH, glyceraldehyde-3-phosphate dehydrogenase; GIP, gastric inhibitory polypeptide; GLP-1, glucagon-like peptide-1.

As is displayed in Figure 4, the stimulation of SGBS adipocytes over 18 h with the incretins GIP and GLP-1 did not significantly affect *CAMP* and *DPP4* gene expression. Of note, GIP induced an elevation in *FABP4* mRNA levels in a dose-dependent manner (Figure 4B), whereas GLP-1 had no effect (Figure 4E), thus suggesting a divergent impact of both incretins on adipocyte lipid metabolism.

## 3. Discussion

The presented data demonstrate for the first time the dynamics of circulating Cathelicidin antimicrobial peptide (CAMP) during an oral glucose tolerance test in a large cohort of adult, insulin-sensitive, and healthy volunteers. The detected range of base-line CAMP serum concentrations was in good accordance with our previous findings in a cohort of morbidly obese patients [12]. Among the metabolically healthy individuals participating in the present study, basal CAMP levels did not exhibit a significant sexual dimorphism—as was recently found in obese individuals [12]—nor a significant impact of overweight and obesity (BMI ≥ 25 kg/m^2^). Importantly, serum CAMP concentrations at the study base-line were negatively correlated with HDL cholesterol and with the adipokine adiponectin, both representing cardioprotective and beneficial metabolic markers [20,21]. This observation is in good accordance with our previous findings in obese patients, where we reported negative correlations between base-line CAMP and HDL cholesterol serum quantities as well as between CAMP and adiponectin gene expression levels in subcutaneous adipose tissue [12]. Of note, the correlation between systemic CAMP and HDL cholesterol levels was apparently not affected by the OGTT in the present study cohort. We furthermore observed a significant positive correlation between systemic CAMP and lipocalin-2 levels which was also maintained throughout the OGTT. Since lipocalin-2, similar to CAMP, is involved in multiple metabolic and inflammatory processes [22], a putative coregulation of these proteins represents an intriguing issue to be addressed by future studies elaborating on the present data.

Alongside CAMP, concentrations of circulating FABP2, FABP4, and DPP4 were investigated. Males exhibited significantly higher base-line DPP4 concentrations than females, while no impact of BMI was observed. FABP4 levels were elevated in women—which is in accordance with findings of a previous study [23]—as well as in overweight individuals in general. In contrast, FABP2 concentrations were not predicted by sex or overweight.

During the OGTT, systemic levels of both fatty acid binding proteins as well as DPP4 exhibited a considerable and significant decline as early as 1 h after glucose ingestion, thus being in good accordance with earlier reports concerning FABP4 [24]. In contrast, we observed a significant rise in systemic CAMP within 2 h of glucose ingestion. This finding suggests a considerable short-term induction of CAMP expression and secretion by insulin, glucose, and/or incretins. Of note, CAMP has previously been reported to be linked to diabetes mellitus type 1 [25], and its circulating levels have been suggested as a marker for diabetic nephropathy [26]. While a recent study reported decreased CAMP serum concentrations in T2D patients [27], results from previously published studies are somewhat incongruent with this observation [28]. Thus, the described impact of raised circulating glucose levels might significantly contribute to our understanding of the interrelation between glucose metabolism and immune-metabolic factors. Of particular interest, our data suggest putative differences in glucose-related systemic CAMP regulation between males and females as well as between normal-weight and overweight individuals. Of note, the observed rise in CAMP quantities appears to be mainly based on effects among the normal-weight subjects. Although substantially elevated base-line triglyceride levels (>200 mg/dL) in a minor proportion of the present study cohort (7 out of 86)—accompanied by elevated CAMP levels in these individuals—might be considered a potential, moderate confounding factor regarding the impact of BMI, our data provide a valid basis for further elaboration on the issue of BMI-related effects on postprandial CAMP kinetics in future approaches.

Given the previously reported role of CAMP in diabetes type 1 [25], as well as the present finding of its significant increase during an OGTT, future studies might involve hyperinsulinemic-euglycemic clamp techniques in order to evaluate CAMP kinetics under conditions of elevated insulin and constant glucose levels. In particular, such approaches might elucidate putative associations of insulin sensitivity and systemic CAMP regulation.

A further question of particular interest arising from the present data is whether the observed systemic CAMP kinetics during the OGTT are specific to glucose ingestion or part of a rather general postprandial regulatory effect being linked to caloric input. As we reported in a very recently published study on CAMP regulation during a 6 h oral lipid tolerance test (OLTT), circulating levels significantly decreased upon the oral ingestion of a carbohydrate- and protein-free lipid preparation, consisting of triglycerides and free fatty acids [29]. Thus, the observed contrary effects of oral glucose and lipid administration might indicate diverging postprandial processes significantly affecting circulating CAMP, thereby arguing for rather nutrient-specific effects. Furthermore, we cannot rule out caloric or dynamic effects. In the present study, the oral glucose ingestion contained about 300 kcal, whereas the lipid solution in the previous study contained about 758.1 kcal. This intriguing issue requires further information and should be addressed by future studies. After glucose ingestion, the short-term effects after 1 and 2 h were investigated due to the rapid increase in serum glucose levels. In comparison, lipid resorption was slower and systemic effects were observed after between 4 and 6 h [29]. Therefore, resorption dynamics might play a role in postprandial CAMP regulation. Future studies are necessary to appoint this observation.

In the current study, we demonstrated an increase in CAMP serum concentrations after oral glucose ingestion for the first time. Yet the source of the increased CAMP amount is unknown so far. Furthermore, the underlying mechanisms of this effect remain to be elucidated. Adipocytes and adipose tissue are sensitive to alterations in carbohydrate homeostasis and have previously been reported as a relevant source of CAMP [9,10]. Therefore, we hypothesized that glucose or glucose-triggered hormones such as insulin, GLP-1, or GIP increase *CAMP* gene expression in adipocytes. In vitro experiments used human adipocytes derived from the established preadipocyte cell line SGBS [30], which has been widely used throughout the last two decades [31]. The morphological phenotype of preadipocytes and mature adipocytes, as well as the gene expression pattern during adipogenic differentiation, are illustrated in Supplementary Figure S2. Of note, SGBS adipocytes exhibited a significant, insulin-dependent decline of *CAMP* mRNA expression under experimental hyperglycemic conditions. Therefore, glucose- and insulin-induced effects in adipocytes apparently do not contribute to the observed elevation of systemic CAMP concentrations after oral glucose uptake. Adipocyte *DPP4* expression levels were reduced by insulin treatment under normoglycemic as well as hyperglycemic conditions in vitro, whereas *FABP4* expression remained unaffected.

Aside from the direct impact of glucose, due to their important role in post-prandial carbohydrate metabolism, the incretins GLP-1 and GIP were considered as putative mediators of *CAMP* induction in adipose tissue. Unlike glucose and insulin treatment, no significant effects of incretins on adipocyte *CAMP* expression levels were observed. Taken together, these findings do not support the hypothesis of the significant contribution of adipocyte gene expression to the metabolically induced elevation of circulating CAMP concentrations. In order to verify this conclusion, additional future studies should apply analogous experimental settings to adipocytes derived from primary stem cells.

While adipocytes might not significantly contribute to metabolite-induced CAMP regulation, this cell type might rather represent a physiological target for CAMP action, particularly in the context of metaflammation and adipose tissue inflammation. Since information on the functional expression of CAMP receptors—such as formyl peptide receptor 2 (Fpr2)—in adipocytes is lacking so far, this important issue should also be addressed by future investigations.

Furthermore, the observed increase during the OGTT might instead be induced by other mediators—such as satiety, gastrin, ghrelin, or further hormones—by the postprandial state per se or by nerval mechanisms.

Since immune cells are also a major source of systemic CAMP, and since high glucose levels have been reported to induce *CAMP* expression in monocytes [32], immune cells might be significantly involved in the observed upregulation of *CAMP*. This intriguing issue should be addressed by future approaches of investigating *CAMP* expression in various immune cell types under hyperglycemic and hyperinsulinemic conditions. In particular, promising experiments should involve the isolation of peripheral blood mononuclear cells (PBMC) during an OGTT and the subsequent quantification of *CAMP* mRNA expression in these cells.

### Limitations

In the present study, we investigated the effects of oral glucose ingestion on FABP2, FABP4, DPP4, and CAMP serum levels. Nonetheless, in real life, meals consist not only of glucose but also of complex carbohydrates, proteins, lipids, and other contents. Therefore, the effect of mixed meals or western diet on FABP2, FABP4, DPP4, and CAMP serum levels might be different. Future approaches applying alternative study designs including hyperinsulinemic-euglycemic clamp techniques might be promising in order to investigate hyperinsulinemic effects at constant glucose concentrations and to correlate adipokine regulation with insulin sensitivity. Out of an initial study cohort of 100 subjects [33], samples of 86 individuals were available for all time points during the OGTT: base-line (0 h), 1 h and 2 h after glucose ingestion. All of these subjects and samples were included in the present investigation in order to ensure the statistical significance of physiologically relevant differences and effects. As we see from the present data, as well as from a previous publication on this study cohort [33], a number of 80–100 examined subjects is sufficient to evaluate the statistical significance of OGTT-induced effects on adipokine kinetics–even if the absolute size of the effect might be rather moderate, as it is the case in the present study.

Adipose tissue contains mature white adipocytes as well as a number of cell types (preadipocytes, immune cells, etc.) summarized as the stromal vascular fraction. In order to investigate if the systemic increase in CAMP levels upon oral glucose ingestion in vivo might be mediated by glucose or glucose-triggered hormones (e.g., insulin, GLP-1, GIP), we conducted in vitro experiments on adipocytes. Differentiated SGBS cells represent a well-established adipocyte model [30,31] applicable for metabolic studies in vitro. White adipocytes are visually characterized by one big lipid vacuole. In SGBS adipocytes, there are many small lipid vacuoles. Therefore, we investigated the phenotype of SGBS adipocytes to ensure their suitability as an appropriate in vitro model of human adipocytes. As is illustrated in Supplementary Figure S2, differentiating SGBS cells exhibit a strong induction of the white adipocyte marker genes, *adiponectin* and *FABP4*, indicating a predominantly white phenotype. A further observed considerable rise in the mRNA levels of the brown/beige adipocyte marker *UCP-1*—due to rosiglitazone-induced PPARγ activity—indicates that differentiated SGBS cells also share some of the metabolic properties of beige adipocytes. This issue has been extensively discussed recently [31]. The adipogenic differentiation of primary adipose-derived stem cells might provide a further appropriate ex vivo adipocyte model which is applicable to verifying the observed effects. Future studies elaborating on the present data and addressing the mechanisms of *CAMP* regulation thus should include cells derived from such primary preadipocytes.

## 4. Materials and Methods

### 4.1. Study Cohort

The present study investigated serum CAMP levels in 86 metabolically healthy adult volunteers who were examined at the University Hospital of Regensburg, Germany, as part of a larger study cohort previously characterized in detail [33]. All individuals gave their informed consent to the study, which was approved by the local ethical committee (protocol code 11-101-0068, 31 March 2011). The cohort included 57 females and 29 males, of which 40 individuals had a normal weight (BMI < 25 kg/m^2^) and 46 were overweight (BMI ≥ 25 kg/m^2^) (all insulin-sensitive with a normal HOMA index). A positive history of any kind of disease, evidence of acute or chronic infection within 10 days prior to the experiment, age <18 years or >55 years, as well as any kind of medication except oral contraceptives, were among exclusion criteria. Furthermore, pregnant and menstruating women were not admitted. Standard anthropometric parameters such as age, BMI, hip circumference, waist circumference, waist/hip ratio, triceps skinfold thickness, and blood pressure were assessed alongside patients’ family history concerning type 2 diabetes and cardiovascular diseases, as well as habits such as smoking and hormonal contraception.

An initial overview of the basic characteristics of the entire study population (n = 100) was provided earlier [33].

### 4.2. Oral Glucose Tolerance Test

The study participants underwent a standard 75-g, 2-h OGTT between 8:00 a.m. and 10:00 a.m. after an overnight fast of 12 h. Venous blood samples were drawn at 0, 60 and 120 min during OGTT and blood serum was prepared via centrifugation (4000 rpm, 5 min).

### 4.3. Quantification of Systemic Proteins and Standard Serum Parameters

Serum protein concentrations were measured in duplicates by ELISA techniques (CAMP: Hycult Biotech, Uden, Netherlands; FABP2, FABP4, DPP4: DuoSet ELISA development systems, R&D Systems, Wiesbaden, Germany) and are given in the text as mean values ± standard deviation. The figures illustrate the means ± standard error of the mean (SEM).

### 4.4. SGBS Cell Culture

Preadipocytes of the cell line SGBS were cultured as described earlier [34]. The human Simpson–Golabi–Behmel syndrome (SGBS) preadipocyte cell strain [30] was kindly provided by Prof. Dr. Martin Wabitsch (University of Ulm, Germany). These primary human cells originate from adipose tissue specimens of a patient suffering from SGBS. Following the provider’s established protocol [30], the cells were differentiated into mature adipocytes within 14 days of culture. SGBS preadipocytes were cultured in DMEM/F12 (1:1) with 10% FCS (purchased from Invitrogen, Darmstadt, Germany). Differentiation into mature adipocytes was induced at 80% confluence of the cells by incubation in serum-free medium, supplemented with 0.01 g/mL transferrin, 20 nM insulin, 0.2 nM triiodothyronine, and 100 nM cortisol (all from Sigma-Aldrich, Deisenhofen, Germany). During the first 4 days of differentiation, the culture medium was additionally supplemented with 2 μM rosiglitazone (BRL 49653) (Cayman, Tallinn, Estonia), 250 μM isobutylmethylxanthine (IBMX), and 25 nM dexamethasone (all purchased from Sigma-Aldrich, Deisenhofen, Germany). The medium was generally replaced every 3–4 days, and cell differentiation was completed after 14 days. The characteristic morphological phenotype of mature adipocytes was controlled and verified by light microscopy.

Mature adipocytes were treated over 18 h with different doses of insulin (0.2 and 2 nM), GIP (10 and 100 nM), and GLP-1 (10 and 100 nM) (all from Sigma-Aldrich, Deisenhofen, Germany). Prior to the presented experiments, all applied doses had been tested in preliminary experiments for potential impact on cell viability. Upon sampling of cell lysates for subsequent RNA preparation and of cell supernatants, lactate dehydrogenase (LDH) activity in cell supernatants was quantified using a colorimetric cytotoxicity detection kit (Roche, Basel, Switzerland) in order to exclude any unintended cytotoxic effects.

### 4.5. Gene Expression Analysis

The total RNA was isolated from mature SGBS adipocytes using the RNeasy^®^ Mini Kit (Qiagen, Hilden, Germany), including DNase digestion (RNase-Free DNase Set, Qiagen, Hilden, Germany). Gene expression was quantified by reverse transcription of 300 ng RNA (QuantiTect Reverse Transcription Kit from Qiagen, Hilden, Germany) and subsequent real-time PCR (RT-PCR) (iTaq Universal SYBR Green Supermix, CFX Connect RT-PCR system; Bio-Rad, Munich, Germany) of the corresponding cDNA. The following primer sequences (forward/reverse) were used:

*Human CAMP*:
5′-TAGATGGCATCAACCAGCGG-3′/5′-CTGGGTCCCCATCCATCGT-3′
*Human DPP4*:
5′-TTCTGCTGAACAAAGGCAATGA-3′/5′-CTGTTCTCCAAGAAAACTGAGCTG-3′
*Human FABP4*:
5′-ATGGGGGTGTCCTGGTACAT-3′/5′-CTTTCATGACGCATTCCACCA-3′
*Human GAPDH*:
5′-GAGTCCACTGGCGTCTTCAC-3′/5′-CCAGGGGTGCTAAGCAGTT-3′

Expression levels of the target gene were normalized to the gene expression of *human glyceraldehyde-3-phosphate dehydrogenase (GAPDH)*. All oligonucleotides used were purchased from Metabion, Martinsried, Germany.

### 4.6. Statistical Analysis

Mean protein concentrations ± standard deviation (SD) were calculated using a statistical software package (SPSS 26.0). Mean values were compared using the Friedman test for related samples and the Mann–Whitney U-test for non-related samples, and the Bonferroni method was used in order to correct for multiple testing. Correlation analysis was performed using the Spearman test for linear variables. Differences with *p*-values less than 0.05 (two-tailed) were considered as statistically significant. In the Figure 2 and Figure S1, mean values are presented and the whiskers show the SEM. In Figure 3, Figure 4 and Figure S2, the results are visualized as box plots, giving the median and the quartile ranges.

## 5. Conclusions

The present study demonstrates the significant and short-term upregulation of circulating Cathelicidin anti-microbial peptide levels following glucose ingestion. While the observed effects might be mechanistically linked to the parameters of glucose metabolism, our data imply that adipocytes are not the site of glucose-, insulin- and/or incretin-induced elevated *CAMP* expression.

## Figures and Tables

**Table 1:** Base-line characteristics of the study population (n = 86) comparing male with female (**A**) and normal weight with overweight individuals (**B**). Mean values ± standard deviation are presented. n.s.: not significant.

A				
Parameters	Study Cohort (n = 86)	Male (n = 29)	Female (n = 57)	*p*
Sex (male/female)	29/57			
Age [y]	26.8 ± 6.1	26.4 ± 5.1	27.0 ± 6.6	n.s.
BMI [kg/m^2^]	25.27 ± 4.94	25.20 ± 4.99	25.31 ± 4.96	n.s.
WHR	0.86 ± 0.08	0.92 ± 0.07	0.83 ± 0.08	<0.001
Glucose [mg/dL]	84.07 ± 12.87	83.72 ± 12.61	84.25 ± 13.11	n.s.
Insulin [mU/L]	10.12 ± 9.15	10.68 ± 13.22	9.84 ± 6.28	n.s.
HOMA-Index	2.14 ± 1.80	2.16 ± 2.43	2.14 ± 1.40	n.s.
Total cholesterol [mg/dL]	197.5 ± 36.7	187.5 ± 39.0	202.6 ± 34.7	n.s.
HDL cholesterol [mg/dL]	65.3 ± 19.1	53.2 ± 11.6	71.5 ± 19.3	<0.001
LDL cholesterol [mg/dL]	109.5 ± 31.8	109.5 ± 36.1	109.5 ± 29.7	n.s.
Triglycerides [mg/dL]	114.2 ± 88.1	126.9 ± 122.5	107.8 ± 64.3	n.s.
CAMP [ng/mL]	28.08 ± 14.45	31.49 ± 15.00	26.34 ± 13.97	n.s.
FABP2 [pg/mL]	1961.07 ± 981.27	1991.11 ± 955.07	1945.79 ± 1002.37	n.s.
FABP4 [pg/mL]	4840.53 ± 4057.25	3982.63 ± 2831.50	5261.68 ± 4503.67	0.016
DPP4 [ng/mL]	487.40 ± 107.62	524.19 ± 103.96	468.68 ± 105.44	0.025
**B**				
**Parameters**	**Lean (n = 40)**	**Overweight (n = 46)**	***p***	
Sex (male/female)	16/24	13/33		
Age [y]	24.65 ± 2.28	28.65 ± 7.46	0.022	
BMI [kg/m^2^]	21.31 ± 1.83	28.72 ± 4.14	<0.001	
WHR	0.83 ± 0.74	0.89 ± 0.08	0.002	
Glucose [mg/dL]	77.45 ± 12.54	89.83 ± 10.19	<0.001	
Insulin [mU/L]	6.89 ± 4.02	12.94 ± 11.26	<0.001	
HOMA-Index	1.45 ± 0.87	2.75 ± 2.16	<0.001	
Total cholesterol [mg/dL]	192.82 ± 36.52	201.63 ± 36.69	n.s.	
HDL cholesterol [mg/dL]	71.35 ± 17.46	60.07 ± 19.09	0.001	
LDL cholesterol [mg/dL]	96.73 ± 27.35	120.57 ± 31.45	<0.001	
Triglycerides [mg/dL]	94.43 ± 45.17	131.43 ± 110.57	n.s.	
CAMP [ng/mL]	25.06 ± 13.15	30.71 ± 15.13	n.s.	
FABP2 [pg/mL]	2073.84 ± 773.68	1863.02 ± 1130.88	n.s.	
FABP4 [pg/mL]	3834.31 ± 4676.08	5667.87 ± 3295.78	<0.001	
DPP4 [ng/mL]	496.18 ± 107.25	479.76 ± 108.53	n.s.	

**Table 2:** Correlations of CAMP serum concentrations with systemic physiological parameters at study base-line.

Parameters Correlated with CAMP	*rho*	*p*
HDL cholesterol	−0.310	0.004
Adiponectin	−0.272	0.011
Angptl4	−0.219	0.043
NT-proANP	+0.270	0.015
FGF-21	+0.243	0.025
Lipocalin-2	+0.296	0.006

## Data Availability

Data concerning the present study are available from the corresponding author upon request.

## Supplementary Materials

The supporting information can be downloaded at: https://www.mdpi.com/article/10.3390/ijms241612901/s1.
