# Supplementary material for: Circulating Concentrations of Cathelicidin Anti-Microbial Peptide (CAMP) Are Increased during Oral Glucose Tolerance Test
Source: Int J Mol Sci. 2023 Aug 17;24(16):12901. doi: 10.3390/ijms241612901 (PMC10454907; doi:10.3390/ijms241612901)
Supplement: Supplementary file 1 [file ijms-24-12901-s001.zip › ijms-2482299-supplementary.pdf]

Supplementary Table S1: Correlation analysis of systemic CAMP concentrations and parameters of glucose and lipid metabolism at study base-line. t = 0: prior OGTT, n.s.: not significant

**Parameters for correlation analysis (t = 0)**	***rho***	***P***
FABP2 (t = 0)	+0.018	n.s.
FABP4 (t = 0)	+0.152	n.s.
DPP4 (t = 0)	-0.092	n.s.
Glucose (t = 0)	+0.023	n.s.
Insulin (t = 0)	+0.092	n.s.

Supplementary Table S2: Correlation analysis of CAMP serum concentrations with adipokines and HDL cholesterol levels after OGTT. t = 2: 2h after OGTT, n.s.: not significant

**Parameters for correlation analysis (t = 2)**	***rho***	***P***
HDL cholesterol (t = 2)	-0.336	0.002
Adiponectin (t = 2)	-0.110	n.s.
Angptl4 (t = 2)	-0.247	0.022
NT-proANP (t = 2)	+0.166	n.s.
FGF-21 (t = 2)	+0.193	n.s.
Lipocalin-2 (t = 2)	+0.257	0.017
FABP2 (t = 2)	+0.032	n.s.
FABP4 (t = 2)	-0.010	n.s.
DPP4 (t = 2)	-0.004	n.s.

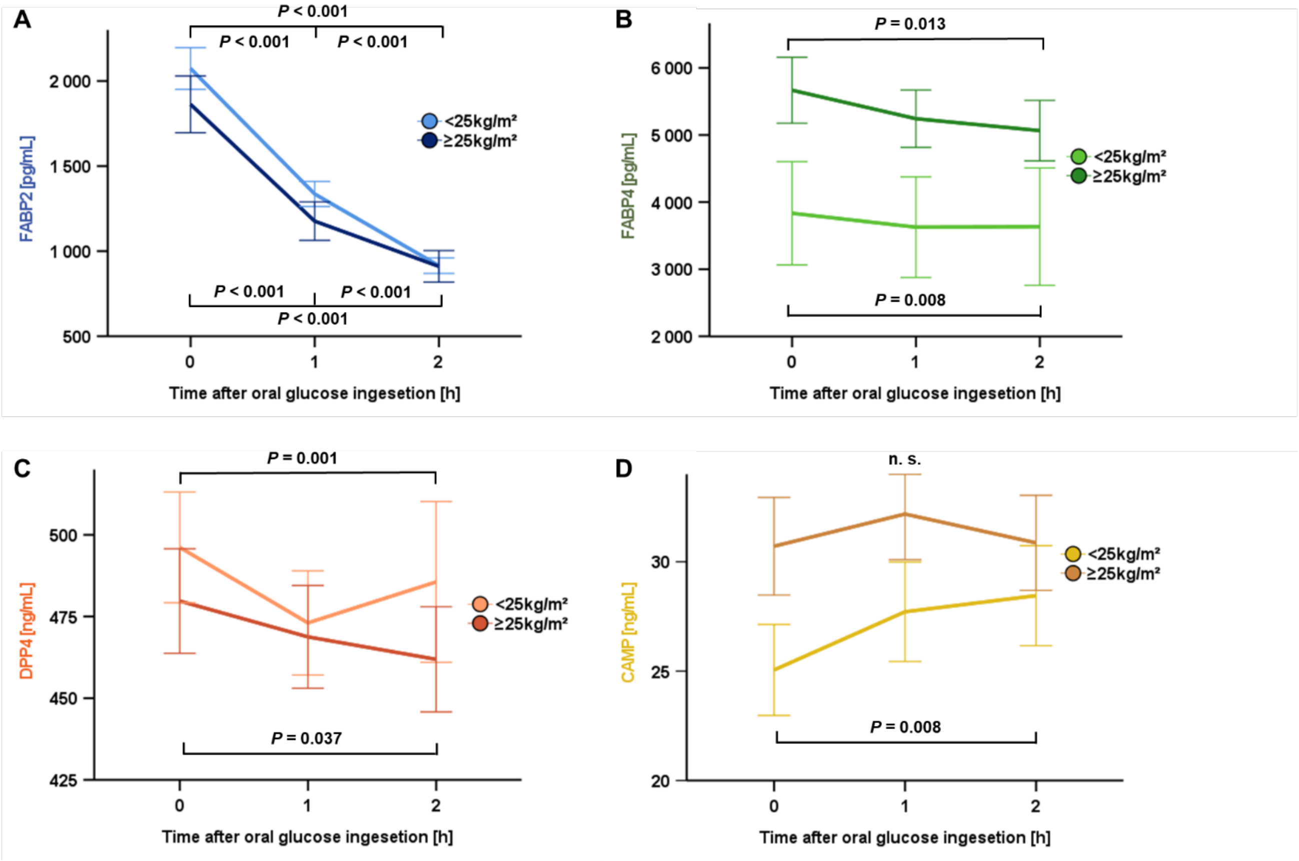

Supplementary Figure S1: Kinetics of FABP2 (A), FABP4 (B), DPP4 (C), and CAMP (D) serum levels during oral glucose tolerance test in normal weight (BMI < 25 kg/m^2; n = 40) and overweight individuals (BMI ≥ 25 kg/m^2; n = 46). n.s.: not significant

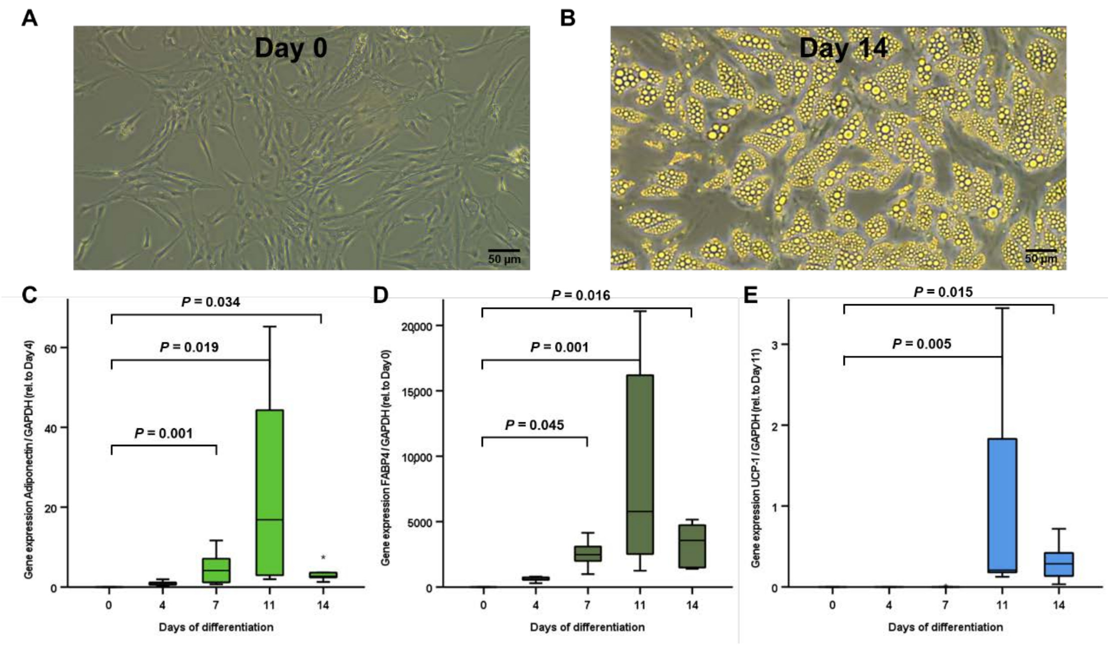

Supplementary Figure S2: Changes in morphology and gene expression in SGBS cells during adipogenic differentiation. Representative light microscopy images of SGBS preadipocytes (A) and mature adipocytes (B) are shown. Gene expression of adiponectin (C), FABP4 (D), and UCP-1 (E) was induced during adipocyte differentiation. N = 5-6; FABP4, fatty acid binding protein 4; UCP-1, uncoupling protein-1. (*: outlier)
